# Association of latent class analysis-derived subphenotypes of acute kidney injury with mortality in critically ill patients with cardiovascular disease: a retrospective cohort study

**DOI:** 10.1186/s12872-022-02587-9

**Published:** 2022-04-07

**Authors:** Yongqing Huang, Zhanchao Xiao, Yong Xie, Shaoxin Zheng, Taihui Yu, Zhixuan Guo, Dan Su, Anqi Song, Yangxin Chen, Shuxian Zhou, Qi Guo, Jingfeng Wang

**Affiliations:** 1 Department of Cardiology, Sun Yat-Sen Memorial Hospital, Sun Yat-Sen University, No. 107 West Yanjiang Road, Guangzhou, 510120 China; 2 Guangdong Provincial Key Laboratory of Arrhythmia and Electrophysiology, Guangzhou, China; 3 Department of Radiology, Sun Yat-Sen Memorial Hospital, Sun Yat-Sen University, Guangzhou, China; 4 Department of Dermatology, Sun Yat-Sen Memorial Hospital, Sun Yat-Sen University, Guangzhou, China; 5 Department of Cardiology, The Second Affiliated Hospital, Xi’an Jiaotong University, Xi’an, China

**Keywords:** Acute kidney injury, Subphenotypes, Latent class analysis, Mortality, Cardiovascular disease

## Abstract

**Background:**

To explore the potential heterogeneity of acute kidney injury (AKI) and evaluate the prognostic differences among AKI subphenotypes in critically ill patients with cardiovascular diseases.

**Methods:**

Data were extracted from the Medical Information Mart for Intensive Care (MIMIC)-III database. Latent class analysis (LCA) was used to explore the potential subphenotypes of AKI in critically ill patients with cardiovascular diseases. The number of classes was identified by the Bayesian information criterion and entropy. The differences in prognostic ability among the AKI subphenotypes were evaluated by logistic regression analysis.

**Result:**

A total of 7738 AKI patients were enrolled in this study. Using LCA, AKI patients were divided into 4 heterogeneous subphenotypes, which were obviously different from the Kidney Disease: Improving Global Outcomes (KDIGO) stages. Interestingly, class 3 classified by LCA was dominated by stage 2, while the mortality rate in class 3 was significantly different from that in class 1 (15.2% vs. 1.6%, *p* < 0.05). After further adjustment, the mortality rate in class 3 remained higher than that in class 1, with an odds ratio of 12.31 (95% confidence interval, 8.96–16.89).

**Conclusions:**

LCA was feasible for AKI classification in critically ill patients with cardiovascular disease, and 4 distinct subphenotypes of AKI patients with different prognoses were identified. Our results highlighted the potential heterogeneity of AKI patients, which is worthy of further investigation.

**Supplementary Information:**

The online version contains supplementary material available at 10.1186/s12872-022-02587-9.

## Introduction

Acute kidney injury (AKI) is a serious clinical event characterized by a sudden decline in renal function, with a 3.2–78% incidence in admission to the intensive care unit (ICU) [1] and a significant correlation with mortality [2]. There is potential heterogeneity in AKI, which leads to complex clinical manifestations and few effective treatments [3]. AKI is also common among patients with cardiovascular disease and is associated with higher mortality in these patients [4, 5]. However, the types of comorbidities and pathophysiological changes in patients with cardiovascular disease were significantly different from those in ICU patients, leading to further heterogeneity of AKI in patients with cardiovascular disease [5, 6]. Whether the prognoses among AKI patients with different clinical features vary remains unknown.

Latent class analysis (LCA), a popular method based on multidimensional data, is used to identify potential heterogeneity among individuals. Emerging studies using LCA to identify the subphenotypes of patients with a single disease, such as metabolic syndrome [7] and obstructive sleep apnea [8], have recently been reported. In the ICU, 2 AKI subphenotypes were identified by LCA, which present different risks for adverse clinical outcomes [9]. However, there is currently no study on the classification of AKI in critically ill patients with cardiovascular disease, and it is unclear whether LCA is applicable to the subphenotype exploration in this group of patients.

In this study, data were obtained from the Medical Information Mart for Intensive Care (MIMIC)-III database, which is composed of a large amount of clinical and test data collected from the ICU [10]. We aimed to evaluate the feasibility of LCA for exploration of AKI subphenotypes in critically ill patients with cardiovascular disease and compare the prognosis among these AKI subphenotypes to provide a theoretical basis for clinical differentiation and prognosis prediction of distinct AKI subphenotypes.

## Methods

### Data source

Related data were extracted from the MIMIC-III database established by Beth Israel Deaconess Medical Center in Boston, Massachusetts, USA [10]. The database includes information on clinical diagnoses and treatments for more than 30,000 patients in the ICU, collected between 2001 and 2012. Information collected in the database includes patient demographics, vital signs, laboratory test results, procedures, medical treatment, clinical records, imaging reports, and patient death events. The use of the MIMIC-III database was approved by the review committee of Massachusetts Institute of Technology and Beth Israel Deaconess Medical Center.

### Study population

**Fig. 1:**
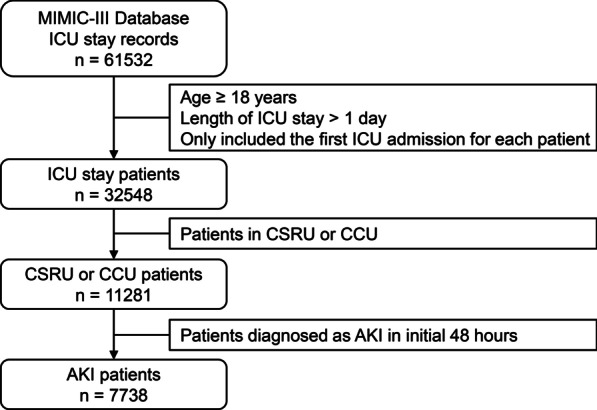
Flow chart of patient selection. Ultimately, 7738 AKI patients in the CCU and CSRU were enrolled in this study. MIMIC-III, Medical Information Mart for Intensive Care-III; ICU, intensive care unit; CSRU, cardiac surgery recovery unit; CCU, cardiac care unit; AKI, acute kidney injury

Adult patients with a length of ICU stay longer than 1 day were included. For patients who were recorded with multiple admissions, only the first ICU admission was extracted. In this study, we focused on patients with cardiovascular disease in the cardiac surgery recovery unit (CSRU) or cardiac care unit (CCU). AKI within 48 h and AKI stage were diagnosed based on the Kidney Disease: Improving Global Outcomes (KDIGO) criteria [11]. Eventually, 7738 AKI patients in the CSRU or CCU were enrolled for the following analysis (Fig. 1).

### Covariates and outcomes

Baseline characteristics were extracted within the initial 24 h after CSRU or CCU admission. The covariates included in this study were as follows: age, sex, body mass index (BMI), unit type, heart rate, respiratory rate, oxygen saturation (SpO_2_), temperature, glucose, systolic blood pressure, diastolic blood pressure, 24-h urine output, use of ventilation, and administration of vasopressors, sedatives, and furosemide.

Comorbidities included coronary artery disease (CAD), atrial fibrillation, congestive heart failure (CHF), hypertension, stroke, sepsis, diabetes, chronic obstructive pulmonary disease (COPD), renal disease, liver disease, and malignancy, which were recorded as International Classification of Diseases, Ninth Revision (ICD-9) codes. Procedures included cardiopulmonary bypass, coronary artery bypass grafting (CABG), and left heart catheterization.

Laboratory test measurements included white blood cell count and levels of hemoglobin, platelets, chloride, sodium, potassium, blood urea nitrogen (BUN), bicarbonate, and creatinine.

Severity at admission was measured by the Sequential Organ Failure Assessment (SOFA) score, Simplified Acute Physiology Score II (SAPS II) score, Elixhauser comorbidity score, and length of ICU stay. The outcome of the current study was 28-day mortality, which was also extracted from the database.

### LCA

LCA was used to explore the potential subphenotype of AKI patients. In this study, baseline characteristics, comorbidities, procedures, and laboratory test measurements, which were described above, were brought into LCA algorithm. The Bayesian information criterion (BIC) criterion, Vuong–Lo–Mendell–Rubin test, and entropy were enrolled to evaluate the proper number of classes. The BIC was a criterion for class number selection, with lower values suggesting model parsimony. Vuong–Lo–Mendell–Rubin test was used to evaluate whether the number of classes provided improved model fit compared to a model using one fewer class. Entropy, an index of how well the classes were separated, ranged from 0 to 1, and values > 0.8 were generally considered a sign of a useful model. LCA was carried out using Mplus software (version 8.5, Muthen & Muthen, Los Angeles, USA).

### Statistics analyses

Continuous variables are presented as the SEM ± SD or median (interquartile range), as appropriate. Categorical variables are presented as numbers (percentages). The chi-square test of categorical variables and analysis of variance or the Kruskal–Wallis test of continuous variables were used for comparisons among groups. In multivariable logistic regression, model 1 was adjusted for age, BMI, and male sex. Model 2 was adjusted for model 1 plus AKI stage. Model 3 was adjusted for Model 2 plus SAPS II score, SOFA score, and Elixhauser comorbidity score. The odds ratio (OR) and 95% confidence interval (CI) values were determined by logistic regression for the prognosis of different classes. Principal component analysis were used to graphically show heterogeneity across AKI classes. DeLong test was used to compare the area under the receiver operating characteristic curve (AUC) values of models. A two-tailed *p* value < 0.05 was considered statistically significant in our study. Statistical analyses were carried out using SPSS (version 23.0, IBM, New York, USA) and the R tool (version 6.3, R Foundation for Statistical Computing, Vienna, Austria).

## Results

### Identification of AKI subphenotypes

**Fig. 2:**
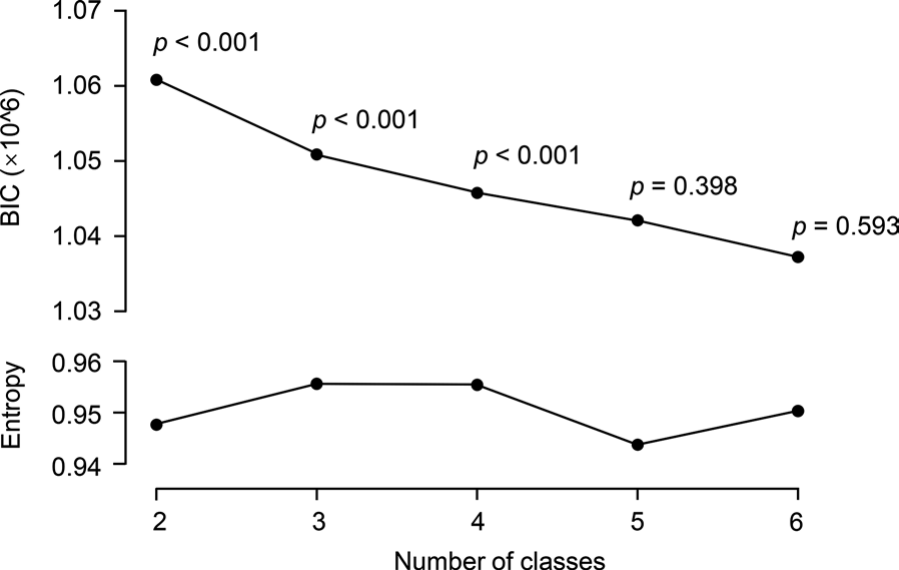
Model fit of the model with different numbers of classes using LCA. The Vuong–Lo–Mendell–Rubin *p*-value was calculated to evaluate whether the number of classes provided improved model fit compared to a model using one fewer class. BIC, Bayesian information criterion; LCA, latent class analysis

The model with 4 classes showed a significantly better model fit than the model with 3 classes (*p* < 0.001), while the model with 5 classes showed no significantly better model fit than the model with 4 classes (*p* = 0.398). The entropy of all models was larger than 0.8, which was the threshold of the useful model. Thus, 4 classes were selected for further analyses (Fig. 2). Then, each patient was assigned to the most likely class by LCA.

### Baseline characteristics of AKI subphenotypes

**Fig. 3:**
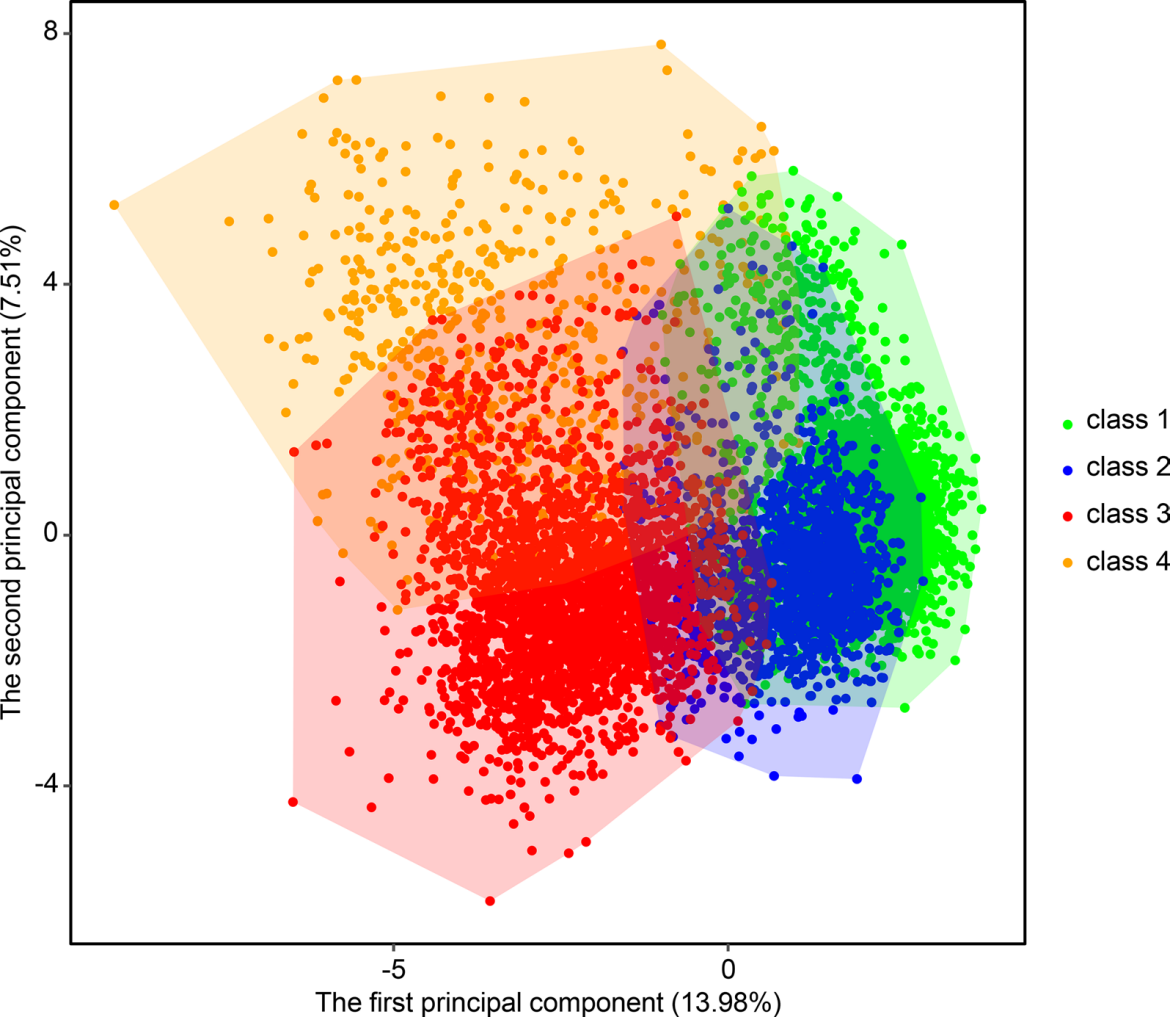
Principal component analysis for LCA-derived AKI classes. Principal component analysis was performed to graphically show heterogeneity across AKI classes. AKI, acute kidney injury; LCA, latent class analysis

There were significant differences in baseline characteristics among the 4 classes (all *p* < 0.05) (Table 1). Principal component analysis showed that class 4 and class 3 were distributed away from class 1 and class 2 (Fig. 3).

**Table 1:** Baseline characteristics of subjects among the 4 LCA-derived AKI classes

Variables	Class 1	Class 2	Class 3	Class 4	*p*
Number	3321	1401	2466	550	
Age, years	68.67 ± 10.54	64.24 ± 14.23	68.68 ± 13.72	70.50 ± 12.59	< 0.001
Male	2462 (74.1)	742 (53.0)	1394 (56.5)	354 (64.4)	< 0.001
Body mass index, kg/m^2^	29.46 ± 5.93	28.75 ± 6.67	29.18 ± 7.41	30.47 ± 8.12	< 0.001
CSRU	3198 (96.3)	1368 (97.6)	523 (21.2)	147 (26.7)	< 0.001
Heart rate, /min	85.63 ± 8.95	83.84 ± 10.98	82.54 ± 16.39	80.29 ± 15.77	< 0.001
Respiratory rate, /min	16.95 ± 2.68	16.95 ± 2.94	19.31 ± 3.80	19.10 ± 3.89	< 0.001
SpO_2_, %	98.04 ± 1.39	98.01 ± 1.33	96.84 ± 2.23	97.03 ± 2.77	< 0.001
Temperature, °C	36.87 ± 0.47	36.85 ± 0.55	36.81 ± 0.67	36.60 ± 0.69	< 0.001
Glucose, mg/dL	132.63 ± 21.00	128.59 ± 20.67	148.59 ± 47.06	149.27 ± 50.64	< 0.001
Systolic blood pressure, mmHg	112.09 ± 8.64	111.97 ± 9.54	115.95 ± 16.45	114.78 ± 17.71	< 0.001
Diastolic blood pressure, mmHg	56.02 ± 6.04	57.54 ± 6.92	60.46 ± 10.57	55.45 ± 11.01	< 0.001
Urine output, mL	1945.00 (1458.50–2636.00)	1840.00 (1305.00–2627.50)	1559.00 (996.75–2404.25)	835.50 (247.25–1589.75)	< 0.001
Ventilation	3285 (98.9)	1386 (98.9)	814 (33.0)	268 (48.7)	< 0.001
Vasopressor	2906 (87.5)	1065 (76.0)	648 (26.3)	265 (48.2)	< 0.001
Sedative	3204 (96.5)	1367 (97.6)	691 (28.0)	226 (41.1)	< 0.001
Furosemide	566 (17.0)	292 (20.8)	228 (9.2)	82 (14.9)	< 0.001
CAD	3302 (99.4)	69 (4.9)	1282 (52.0)	253 (46.0)	< 0.001
Atrial fibrillation	1428 (43.0)	658 (47.0)	866 (35.1)	231 (42.0)	< 0.001
CHF	817 (24.6)	384 (27.4)	1226 (49.7)	364 (66.2)	< 0.001
Hypertension	264 (7.9)	51 (3.6)	220 (8.9)	287 (52.2)	< 0.001
Stroke	207 (6.2)	75 (5.4)	197 (8.0)	27 (4.9)	0.002
Sepsis	278 (8.4)	218 (15.6)	731 (29.6)	293 (53.3)	< 0.001
Diabetes	1326 (39.9)	197 (14.1)	739 (30.0)	289 (52.5)	< 0.001
COPD	317 (9.5)	137 (9.8)	406 (16.5)	91 (16.5)	< 0.001
Renal disease	270 (8.1)	52 (3.7)	248 (10.1)	289 (52.5)	< 0.001
Liver disease	32 (1.0)	27 (1.9)	56 (2.3)	31 (5.6)	< 0.001
Malignancy	124 (3.7)	115 (8.2)	287 (11.6)	48 (8.7)	< 0.001
Cardiopulmonary bypass	3150 (94.9)	1089 (77.7)	267 (10.8)	77 (14.0)	< 0.001
CABG	3164 (95.3)	2 (0.1)	242 (9.8)	62 (11.3)	< 0.001
Left heart catheterization	1435 (43.2)	177 (12.6)	1143 (46.4)	146 (26.5)	< 0.001
White blood cell count, × 10^9^/L	13.16 ± 5.15	13.08 ± 6.19	12.27 ± 6.03	12.78 ± 7.30	< 0.001
Hemoglobin, g/dL	9.84 ± 2.04	9.93 ± 1.93	11.72 ± 1.99	10.03 ± 1.63	< 0.001
Platelet, × 10^9^/L	164.38 ± 60.03	161.50 ± 67.91	239.12 ± 101.52	219.03 ± 100.62	< 0.001
Chloride, mg/dL	108.09 ± 3.96	108.65 ± 4.16	103.54 ± 5.16	101.58 ± 6.78	< 0.001
Sodium, mg/dL	136.47 ± 2.78	137.52 ± 3.36	138.19 ± 4.23	136.30 ± 5.40	< 0.001
Potassium, mg/dL	4.55 ± 0.82	4.34 ± 0.82	4.10 ± 0.65	4.69 ± 0.87	< 0.001
BUN, mg/dL	17.49 ± 7.50	16.53 ± 7.00	23.20 ± 10.99	68.05 ± 23.68	< 0.001
Bicarbonate, mg/dL	23.60 ± 2.39	23.36 ± 2.81	24.49 ± 4.61	22.00 ± 5.33	< 0.001
Creatinine, mg/dL	0.80 (0.70–1.00)	0.80 (0.60–1.00)	1.00 (0.80–1.40)	3.30 (2.50–5.10)	< 0.001

Continuous variables are presented as the SEM ± SD or median (interquartile range), as appropriate. Categorical variables are presented as numbers (percentages). The chi-square test of categorical variables and analysis of variance or Kruskal–Wallis test of continuous variables were used for comparisons among groups. AKI, acute kidney injury; LCA, latent class analysis; CSRU, cardiac surgery recovery unit; CAD, coronary artery disease; CHF, congestive heart failure; COPD, chronic obstructive pulmonary disease; CABG, coronary artery bypass grafting; BUN, blood urea nitrogen

Both class 1 and class 2 were dominated by CSRU patients, and class 1 was more likely to be male (74.1%) and have CAD (99.4%). Patients in class 2 were the youngest (64.24 ± 14.23 years) and least likely to have diabetes (14.1%), CABG (0.1%), and left heart catheterization (12.6%). Class 4 was dominated by hypertension (52.2%), renal disease (52.5%), and CHF (66.2%), which was characterized by the highest creatinine level and the lowest urine output. Compared with the other 3 classes, class 3 had the highest diastolic blood pressure and the lowest use rate of ventilation (33.0%), vasopressors (26.3%), and sedatives (28.0%).

### Comparison of AKI stages and disease severity among the 4 AKI classes

The distribution of AKI stages differed among the 4 AKI classes. The majority of patients in class 1, class 2, and class 3 were stage 2 patients. Class 4 was dominated by stage 3 patients (53.1%) (Table 2). The SAPS II score, SOFA score, and Elixhauser score were highest in class 4 (*p* < 0.001) (Table 3).

**Table 2:** Association between AKI stage and LCA-derived AKI classes

AKI stage	Class 1	Class 2	Class 3	Class 4	*p*
Stage 1	1205 (36.3)	455 (32.5)	694 (28.1)	106 (19.3)	< 0.001
Stage 2	1912 (57.6)	812 (58.0)	1379 (55.9)	152 (27.6)	< 0.001
Stage 3	204 (6.1)	134 (9.6)	393 (15.9)	292 (53.1)	< 0.001

Data were presented as the number (percentage). The chi-square test was used for comparison among groups. AKI, acute kidney injury; LCA, latent class analysis

**Table 3:** Association of disease severity score and outcomes with LCA-derived AKI classes

	Class 1	Class 2	Class 3	Class 4	*p*
SAPS II score	37.14 ± 11.85	35.08 ± 13.08	35.66 ± 13.10	48.81 ± 14.03	< 0.001
SOFA score	5.00 (3.00–7.00)	5.00 (3.00–7.00)	3.00 (2.00–5.00)	6.00 (5.00–9.00)	< 0.001
Elixhauser comorbidity score	0.00 (0.00–5.00)	1.00 (0.00–6.00)	3.00 (0.00–8.00)	7.00 (3.00–12.00)	< 0.001
Length of ICU stay, days	2.23 (1.29–3.92)	2.27 (1.32–4.38)	3.02 (1.90–5.68)	4.07 (2.16–7.33)	< 0.001
28-day mortality	52 (1.6)	66 (4.7)	375 (15.2)	138 (25.1)	< 0.001

Continuous variables are presented as the SEM ± SD or median (interquartile range), as appropriate. Categorical variables are presented as numbers (percentages). The chi-square test of categorical variables and analysis of variance or the Kruskal–Wallis test of continuous variables were used for comparisons among groups. AKI, acute kidney injury; LCA, latent class analysis; SAPS II, Simplified Acute Physiology Score II; SOFA, Sequential Organ Failure Assessment

### Differences in prognosis among the 4 AKI classes

Patients in class 4 had the highest 28-day mortality rate (25.1%), followed by those in class 3 (15.2%). Additionally, the length of ICU stay was the longest in class 4 (*p* < 0.001) (Table 3). In univariate model, class 4 showed the highest risk of 28-day death with an OR of 21.06 (95% CI 15.06–29.44) (Additional file 1: Table S1). After adjustment in model 3, class 3 showed the highest risk of 28-day death with an OR of 16.21 (95% CI 11.83–22.18), followed by class 4 with an OR of 8.31 (95% CI 5.75–12.02) (Table 4).

**Table 4:** Prognostic difference among the 4 AKI classes by multivariable logistic regression

	OR	95% CI	*p*
*Model 1*			
Class 1	Reference		
Class 2	3.29	2.27–4.79	< 0.001
Class 3	10.97	8.15–14.77	< 0.001
Class 4	20.37	14.54–28.53	< 0.001
*Model 2*			
Class 1	Reference		
Class 2	3.03	2.09–4.14	< 0.001
Class 3	9.29	6.88–12.54	< 0.001
Class 4	10.72	7.52–15.30	< 0.001
*Model 3*			
Class 1	Reference		
Class 2	2.98	2.03–4.36	< 0.001
Class 3	16.21	11.83–22.18	< 0.001
Class 4	8.31	5.75–12.02	< 0.001

Multivariable logistic regression was used to evaluate the association between 28-day mortality and AKI classes, in which class 1 was used as a reference. Model 1 was adjusted for age, body mass index, and male sex. Model 2 was adjusted for model 1 plus AKI stage. Model 3 was adjusted for Model 2 plus SAPS II score, SOFA score, and Elixhauser comorbidity score. AKI, acute kidney injury; OR, odds ratio; CI, confidence interval; SAPS II, Simplified Acute Physiology Score II; SOFA, Sequential Organ Failure Assessment

### Comparison of prognostic prediction ability between AKI stages and AKI classes

**Fig. 4:**
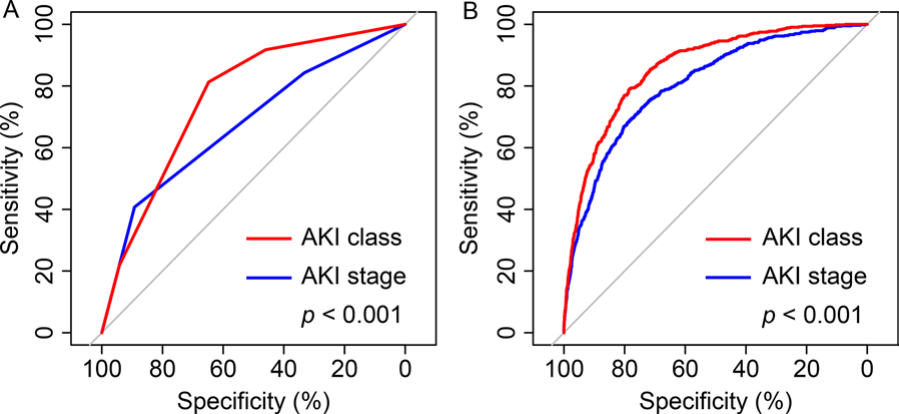
Comparison of ROC curves. **A** Red line indicated model using AKI class. Blue line indicated model using AKI stage. **B** Red line indicated model using AKI class and adjusted variables. Blue line indicated model using AKI class and adjusted variables. Adjusted variables included age, body mass index, male sex, SAPS II score, SOFA score, and Elixhauser comorbidity score. DeLong test was used to compare the AUC values of models. ROC, receiver operating characteristic; AKI, acute kidney injury; SAPS II, Simplified Acute Physiology Score II; SOFA, Sequential Organ Failure Assessment; AUC, area under the ROC curve

In unadjusted model, model using AKI classes (AUC, 0.762, 95% CI 0.745–0.778) showed a significant higher AUC value than model using AKI stages (AUC, 0.678, 95% CI 0.657–0.700, *p* < 0.001). After adjustment, model using AKI classes (AUC, 0.860, 95% CI 0.845–0.874) also showed a significant higher AUC value than model using AKI stages (AUC, 0.806, 95% CI 0.788–0.823, *p* < 0.001) (Fig. 4).

## Discussion

This study extracted clinical data and prognosis information for 7738 critically ill AKI patients with cardiovascular disease from the MIMIC-III database. The potential heterogeneity of AKI was well recognized, but it was unknown that how to classify these AKI patients using the high-dimension clinical data. Moreover, the number as well as the clinical value of AKI subphenotypes were also unknown. Using LCA, 4 distinct AKI classes with remarkable heterogeneity were identified, and each of them had specific clinical traits. Interestingly, these new AKI classes were associated with mortality, and even had advantages in predicting prognosis compared with traditional AKI stages. To the best of our knowledge, this is the first study to use LCA to mine the potential subphenotypes of AKI in patients with severe cardiovascular disease. Our study has 3 new findings. First, 4 AKI classes were identified in patients with severe cardiovascular disease, highlighted the remarkable AKI heterogeneity in these population. Second, our study demonstrated that LCA method was feasible for mining the potential subphenotypes of AKI, raising the possibility of LCA on data mining in clinical researches. Third, these novel AKI classes showed different clinical features and better prognostic prediction ability than traditional AKI stages, which might help in clinical prognosis evaluation.

AKI is a clinical syndrome with high incidence and mortality in the ICU. The classification and prognosis assessment of AKI were mainly based on the KDIGO criteria, which include only creatinine level and urine output [11]. Our novel AKI classes were identified using clinical data and showed differences with AKI stages. Class 1 was mainly CAD patients who had undergone CABG, and thus, the pathophysiology of AKI might include cardiopulmonary bypass-induced hemodilution [12], renal ischemia reperfusion [13], and systemic inflammatory response [14]. Class 2 was mainly patients with AF, who had the highest use rate of ventilation, sedatives, and furosemide. In addition, the rate of cardiopulmonary bypass among these patients was 77.7%. AKI in class 2 might be induced by acute tubular necrosis and embolic events caused by hemodynamic instability [15] and anticoagulation-related nephropathy [16]. Class 4 was similar to stage 3 by the KDIGO criteria, which is characterized by the highest creatinine level and lowest urine output with the highest 28-day mortality. After further adjustment, the risk of 28-day mortality remained significant compared with class 1 mortality. In addition, the mortality rates of class 1, class 2, and class 4 increased with the gradual increase in creatinine level, which was also consistent with the regularity of KDIGO stage classification. Not only did the three classes have their own characteristics, but the prognoses according to LCA and KDIGO stages were consistent. However, the 28-day mortality of class 3, which was also dominated by stage 2, was significantly higher than that of class 1 and class 2. Herein, class 3 might be a novel subphenotype that could not be easily identified by traditional AKI criteria.

Class 3 was dominated by patients in the CCU, and the proportion of CAD patients was 52.0%. The proportion of left heart catheterization was the highest. In addition, patients in class 3 were less likely to have hypertension and diabetes, but their blood pressure and glucose level were significantly higher than those in class 1 and class 2 after admission, indicating that the increase in blood pressure and glucose level might be associated with the stress response. It has been reported that glucose oxidation is enhanced in CAD patients under stress, which could further prevent insulin secretion [17]. AKI in CAD patients after left cardiac catheterization might be associated with contrast nephropathy [18] and ischemic nephropathy [19].

Notably, LCA was used to explore AKI subphenotypes in critically ill patients with cardiovascular disease. LCA tends to be a popular method applied to clinical datasets to identify subphenotypes of diseases [20]. Both BIC criteria and entropy demonstrated that it was reasonable to divide AKI patients into 4 classes. Only variables extracted in the initial 24 h following admission were entered into LCA, while disease severity scores were excluded. Interestingly, the LCA-derived subphenotypes showed a significant association with 28-day mortality. Our results supported that LCA allowed a more nuanced understanding of AKI heterogeneity and their effects on outcomes.

There were several limitations should be mentioned. First, AKI was diagnosed within 48 h of admission according to the KDIGO criteria. We aimed to diagnose and classify AKI patients as rapidly as possible, which could help for risk assessment and clinical decision-making. Thus, we enrolled AKI patients diagnosed within 48 h. Nevertheless, AKI with an onset after 48 h was not considered in our study, which might result in inevitable bias. Second, all the comorbidities were recorded as ICD-9 codes, which might not satisfy the latest diagnostic criteria for some diseases. Third, there was selection bias because of the retrospective study design. Thus, future external validation will help enhance the credibility of our results.

## Conclusion

In conclusion, LCA was feasible for AKI classification in critically ill patients with cardiovascular disease, and 4 distinct subphenotypes of AKI patients with different prognoses were identified. Our results highlighted the potential heterogeneity of AKI patients, which is worthy of further investigation.

## Supplementary Information

**Additional file 1: Table S1.** The association between each variable and 28-day mortality by univariate logistic regression.

## Data Availability

Data in this study were obtained from the Medical Information Mart for Intensive Care (MIMIC-III) database (https://physionet.org/content/mimiciii/1.4/, December 23, 2021), which is a freely accessible critical care database contained large number of clinical and trial data from the real world.

## Abbreviations

AKI: Acute kidney injury
AUC: Area under the receiver operating characteristic curve
BMI: Body mass index
BIC: Bayesian information criterion
BUN: Blood urea nitrogen
CCU: Cardiac care unit
CSRU: Cardiac surgery recovery unit
CAD: Coronary artery disease
CHF: Congestive heart failure
CABG: Coronary artery bypass grafting
COPD: Chronic obstructive pulmonary disease
CI: Confidence interval
ICU: Intensive care unit
ICD-9: International Classification of Diseases: Ninth Revision
KDIGO: Kidney Disease: Improving Global Outcomes
LCA: Latent class analysis
MIMIC: Medical Information Mart for Intensive Care
OR: Odds ratio
SpO_2_: Oxygen saturation
SOFA: Sequential Organ Failure Assessment
SAPS II: Simplified Acute Physiology Score II
